# Olecranon Bursitis, Beau's Lines, Biett's Collarettes, and Crown of Venus

**DOI:** 10.4269/ajtmh.16-0386

**Published:** 2017-02-08

**Authors:** Walter de Araujo Eyer-Silva, Guilherme Almeida Rosa da Silva, Fernando Raphael de Almeida Ferry

**Affiliations:** 1 Hospital Universitário Gaffrée e Guinle, Centro de Ciências Biológicas e da Saúde, Universidade Federal do Estado do Rio de Janeiro (UNIRIO), Rio de Janeiro, Brazil.

A 67-year-old human immunodeficiency virus (HIV)–infected Brazilian male patient presented with a generalized nonpruritic, confluent papulosquamous and nodular rash, first noted 7 days previously (Figure 1

**Figure 1.**
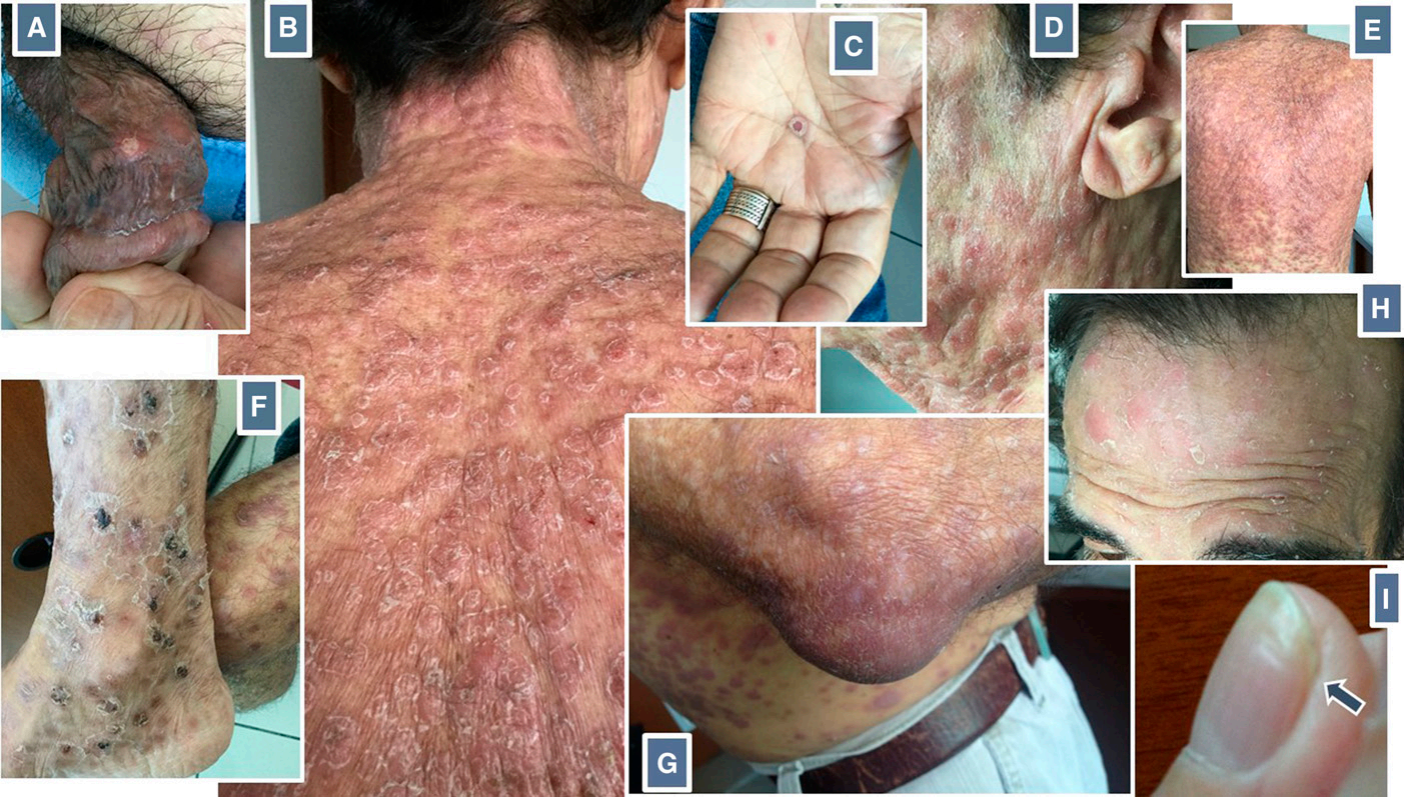
(**A**) A painless healing chancre on penis shaft; (**B**, **D** and **E**) generalized papulosquamous and nodular rash leaving no spared area on the back; (**C**) “collarette of Biett” as a feature of the palmar rash: papules surrounded by a ring of scale; (**F**) ulcero-necrotic lesions with a scale-crust, resembling malignant syphilis; (**G**) olecranon bursitis presenting as an asymptomatic, unilateral, fluctuant swelling at the tip of the right elbow; (**H**) “crown of Venus” (corona veneris) bordering the scalp: scaling papular lesions around the hairline; (**I**) transverse depression (Beau's line) approaches the distal edge of a thumb nail (arrow). Images were taken on the 18th (**A**, **B**, **C**, **D**, **E**, **F**), 25th (**H**), 34th (**G**), and 70th (**I**) day after the rash was first noted.

). The rash was so widespread that there was no spared area on the back. Some lesions around the ankles had a necrotic aspect with scale crust. There were no systemic symptoms or mucosal lesions. A painless healing chancre was evident on the penis shaft.

The patient was on successful antiretroviral therapy for more than 15 years. The CD4 cell count was 666 cells/mm^3^. Plasma HIV viral load measurements were consistently below detection limits. Previous tests were negative for syphilis. Novel studies showed a Venereal Disease Research Laboratory titer of 1:256 and fluorescent treponemal antibody absorption reactive for IgG and IgM. A diagnosis of secondary syphilis was made. It is known that HIV-infected patients with secondary syphilis are more likely to present with a concomitant genital ulcer.^1^ Skin lesions slowly regressed after three consecutive weekly administrations of 2.4 million units of intramuscular benzathine penicillin G. No Jarisch–Herxheimer reaction occurred.

While on treatment, the spectrum of syphilis manifestations continued to unfold. A crown of Venus (corona veneris) rash was recorded in the 25th and a right olecranon bursitis on the 34th day after the start of the rash. Slowly and distally moving symmetric transverse depressions of the nail plate (Beau's lines), more evident on thumb nails, were observed over several weeks. After treatment, a complete remission of all tegumentary abnormalities was observed.

Secondary syphilis results from hematogenous and lymphatic dissemination of treponemes. It may present in a myriad of ways, involve virtually any organ system, and mimic a wide range of other diseases.^2^ HIV infection may alter the clinical course and the presentation of syphilis.^3^

Syphilitic bursitis and Beau's lines are long recognized features of secondary syphilis, but few case descriptions are available on modern electronic databases.^4^,^5^ Syphilitic bursitis most often affects the olecranon bursa in men, but almost any bursa may be involved.^4^ Previous trauma may play a role. Patellar syphilitic bursitis used to be recorded in patients employed as cleaners.^4^ Beau's lines result from temporary arrest of proximal nail matrix proliferation and may be a result of any serious systemic insult.

The wide spectrum of usual and unusual manifestations of what Sir William Osler coined “the great imitator” needs always to be kept in mind by both tropical doctors and clinicians of all specialties. Informed consent of the patient was obtained for publication of the case.

## References

[1] Rompalo AM, Lawlor J, Seaman P, Quinn TC, Zenilman JM, Hook EW (2001). Modification of syphilis genital ulcer manifestations by coexistent HIV infection. Sex Transm Dis.

[2] Dourmishev LA, Dourmishev AL (2005). Syphilis: uncommon presentations in adults. Clin Dermatol.

[3] Balagula Y, Mattei PL, Wisco OJ, Erdag G, Chien AL (2014). The great imitator revisited: the spectrum of atypical cutaneous manifestations of secondary syphilis. Int J Dermatol.

[4] Thomas EW, Rook AJ (1949). Syphilitic bursitis with report of a case. Lancet.

[5] Cormia FE (1938). Syphilitic onychia: report of case which ungual changes helped to establish the diagnosis and the time of syphilitic infection. Arch Derm Syphilol.

